# Exploring the impact on contact adhesion layer properties in numerical simulations

**DOI:** 10.1371/journal.pone.0312436

**Published:** 2024-10-28

**Authors:** Reza Shamim

**Affiliations:** School of Aeronautics, Northwestern Polytechnical University, Xi’an, Shaan’xi, China; TU Dublin Blanchardstown Campus: Technological University Dublin - Blanchardstown Campus, IRELAND

## Abstract

This paper presents a comprehensive investigation into the impact of key parameters on contact adhesion layer properties using numerical simulations, addressing fundamental questions in contact mechanics. Aiming to explore interfacial penetration and contact pressure dynamics between a wavy punch and an adhesive-coated body, the study focuses on the influence of adhesive layer thickness, elasticity modulus, and punch geometry on mechanical behavior. The study includes the application of Green’s function to address deficiencies in existing models, revealing how contact stiffness, influenced by the flexibility relationship between the coating and substrate, affects the size of the contact area. Finally, conclusions are drawn that adjusting coating factors can induce full contact conditions. Quantitative analysis shows a 2.23-fold increase in load-bearing capacity with a 2 mm increase in adhesive layer thickness, and a 23-fold increase with a toughness ratio rise from 0.1 to 5. These findings are recommended for optimizing adhesive layer properties, contributing to advancements in materials science and innovation.

## Introduction

A subfield of mechanics known as contact mechanics examines the deformation of solids that come into contact with each other at one or more points [1–4]. Homogeneous bodies have been the central focus of research in contact mechanics, though recent studies are increasingly addressing the complexities of composite and variable-density materials [5–10]. To overcome contact mechanics difficulties involving homogeneous materials, regarding, several theoretical models [11–15] and numerical approaches [16–20] have been provided in the previous several decades. Nonetheless, interest in solving problems related to nonhomogeneous bodies, especially layered (multi-layered) systems, has been growing [21–24], with pioneering contributions from Argatov [25–27]. This shift reflects the increasing recognition of the practical significance and complex behavior of such materials in various engineering applications. A thin layer covering the top of a bulk material with varied mechanical characteristics is common in cases such as coated substrates, oxidized metals, and painted components. This configuration often enhances the surface properties, providing improved durability, corrosion resistance, and aesthetic appeal. Comprehending the behavior of contact between layered materials is essential for various applications, like rubber seals, which typically use a silicone layer to lower friction, or gears that consist of two rubber sheets with distinct mechanical characteristics to increase the overall stiffness and viscoelastic friction of them.

Additionally, to investigate the biological materials and tissues a number of layered inherent substances are presented [28]. For instance, many studies [29, 30] have shown the existence of at least two layers with distinct mechanical properties concerning, human skin. These layers are the outer skin layer and the underlying tissues, with elastic moduli that can differ from each other. The dimensional scale of the contact has a significant influence on how the layered structure of some materials affects the behavior of the contact. It is well-established that in this case, the volume of the deformed region has a linear behavior that roughly corresponds to the zones of contact area, greatly influencing the total mechanical characterization of the substance [31, 32].

Consequently, no appreciable distinctions would be observed between the contact behavior of a layered matrix and a homogeneous body when considering macroscopic smooth contacts, specifically in cases where the half-space thickness is significantly larger than the adhesive material thickness. Alternatively, when the half-space and adhesive layers have equal thickness, in fact, surfaces are usually somewhat rough due to length-scale variations spanning several orders of magnitude. The ultimate contact behavior will be highly dependent on the unique mechanical and geometrical features of the bulk substrate and layer in such a situation, making the layered system no longer predictable as a homogenous entity. To represent the cohesive behavior of layered materials, various methods have been suggested.

The model provided by Bec et al. highlights a new method combining nanoindentation experiments and imaging procedures, which accurately determines the actual tip-sample contact area and measures contact stiffness against penetration depth, thereby addressing challenges in interpreting nanoindentation tests, particularly for surfaces with local roughness and heterogeneity [33, 34]. Comparably, the Duc et al. model [35] determines the equivalent compliance of the layered solid by combining the individual compliances of the constituents, appropriately weighted using particular functions of the layer thickness, contact area size, and Poisson’s ratios. By using a corrective function, whose functional form is determined by dimensional analysis and whose coefficients are adjusted using finite element computations, Hærvig et al. [36] proposed a model to apply the Johnson–Kendall–Roberts (JKR) theory to the case of layered materials [37]. While these models are useful for qualitative evaluations, they become less accurate when the layered system is reduced to a half-space at small scales, especially when the adhesive layer’s thickness exceeds that of the half-space body. Moreover, these models suit single asperity contacts better than rough contact analysis since they are often grounded on the Hertzian and JKR theories. Another work studied the numerical problem of a sliding viscoelastic cylinder with adhesion, showing the friction coefficient depends on the Maugis–Tabor parameter, dimensionless load, speed, and modulus ratio, with significant adhesion effects at higher parameter values [38]. It also modeled the rolling behavior of a rigid cylinder on an inclined rubber plane, finding qualitative agreement with experiments for large Maugis–Tabor parameters, with friction force increasing linearly at low velocities and decaying at high speeds, aligning with the Persson–Brener theory for two power law regimes, while small Maugis–Tabor values complicate the relationship with cut-off stress [39]. The viscoelastic material behavior under the force of a rigid flat punch characterization had shown that detachment has a simple behavior between Kendall’s elastic solutions and the cohesive strength limit, with minimal dependence on loading process details, and pull-off force peaking at high unloading speeds where energy dissipation is negligible [40].

A comprehensive exploration is conducted, aimed at elucidating numerical analyses and simulations. The focus is on investigating the impact of key parameters in contact problems, specifically the interfacial penetration and contact pressure induced by a wavy punch interacting with a body coated with an adhesive material. The importance of this paper lies in its enhancement of contact mechanics understanding by revealing how optimizing adhesive layer properties can significantly improve load-bearing capacity and contact conditions. Despite extensive research in contact mechanics, several gaps remain in understanding the behavior of layered materials, particularly when scaled to smaller dimensions or subjected to variations in adhesive layer properties. Existing models often struggle with accuracy when the adhesive layer is comparable in thickness to the bulk material or when dealing with rough, non-homogeneous contacts. Many current approaches, such as those by Bec et al. and Duc et al., focus on qualitative assessments and specific contact scenarios, often falling short in general applicability or precision under varying conditions. The novelty of this work lies in the proposal of an alternative approach solution to the issue of a stiff wavy profile compressed in contact with an elastic substrate coated with an elastic layer, departing from the basic solution established in the wavevector domain by Almqvist in Ref. [12]. The highlight of the present work is to provide a detailed understanding of the intricate dynamics involved in such contact conditions. The overarching aim is to investigate the influence of varying the mentioned parameters, on the mechanical behavior of coated structures through numerical simulations. This investigation seeks to optimize adhesion performance for practical applications by understanding how these factors interact and affect the overall performance of the system. The approach relies on the development of Green’s function, which links stresses with interfacial displacements. In the end, one of the related works [41] is contrasted with the outcomes and the method used, including the calculation of Young’s modulus and corrections for contact area and penetration, leading to reliable values of hardness and reduced Young’s modulus.

## Materials and methods

**Fig 1:**
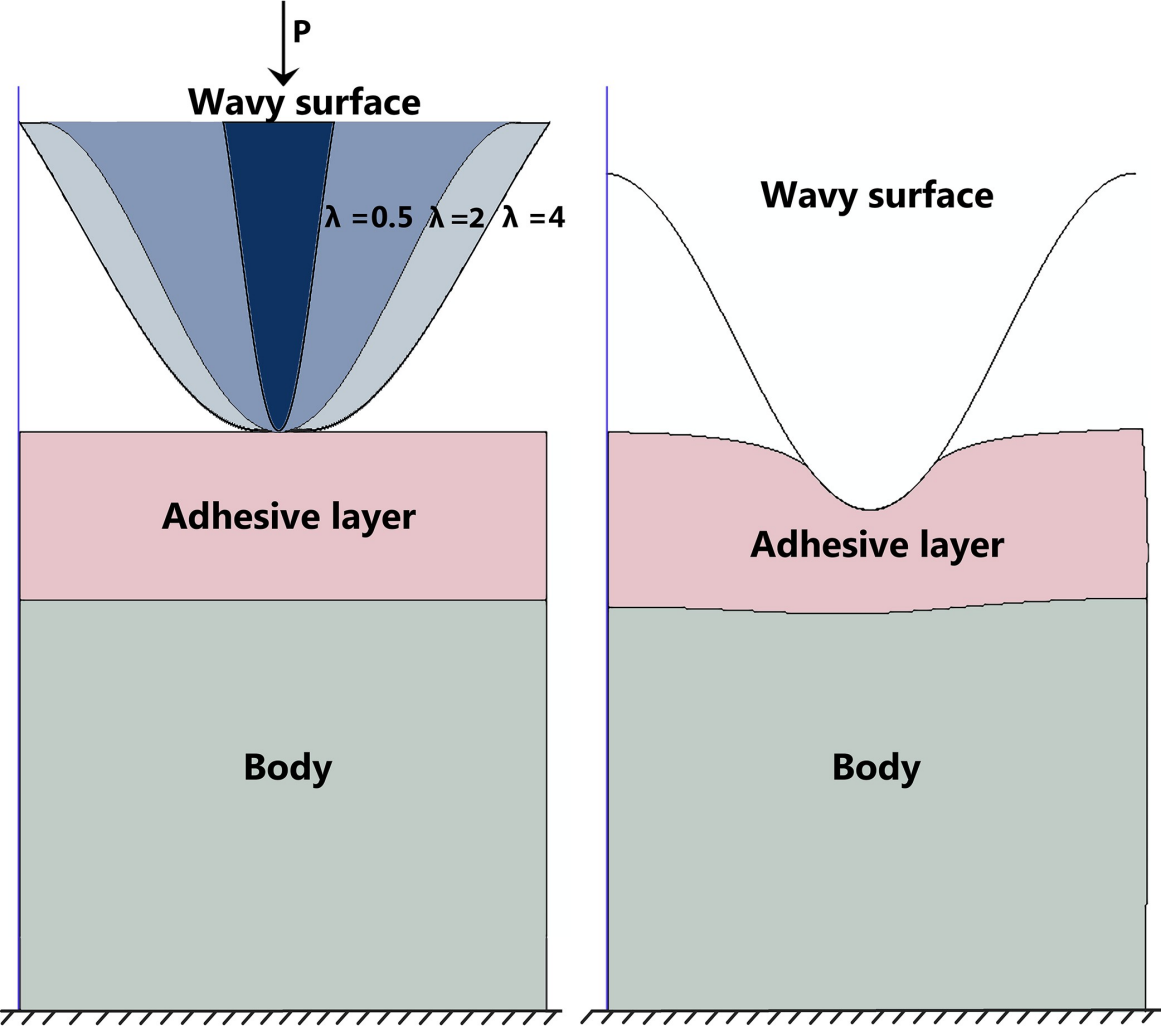
The schematic of the problem.

To address the research problem, numerical simulations are conducted. The methodology involves employing mathematical formulations, including the linear theory of viscoelasticity and expressions for surface displacements, to analyze the behavior of the coated structure under varying conditions. In order to use Hooke’s law to solve the problem, it is assumed that the elastic body is homogeneous and isotropic. The study employs specific boundary conditions to simulate the mechanical test condition. In conducting the finite element analysis of the structures using Abaqus, various conditions were implemented to facilitate the simulation process. The computer-aided design models of the structure were fixed at the bottom, with the adhesive layer applied on top. The entire structure was then subjected to a rigid co-sinusoidal punch that followed a specific function. The analysis utilized a dynamic solver, and results from the numerical simulations were obtained using built-in models. A general surface-to-surface contact was modeled using a finite-sliding formulation, allowing for relative motion between interacting surfaces. For normal interaction, a hard contact approach was employed to prevent surface penetration, ensuring accurate physical representation. Tangential behavior was defined by a penalty friction formulation with a specified coefficient of 0.24, permitting sliding when the shear stress exceeded the friction threshold. The material properties utilized in the simulation consist of a variable relationship between the modulus of elasticity of half-space to adhesive. Several assumptions were made, including a symmetric, uniform in-plane strain tensor E, where in-plane displacements and stresses were considered uniform through the thickness, with no out-of-plane strain. The normal stress was non-zero, while shear stress components were negligible. To simplify the movement of the indenter, which was assumed to be rigid, the upper plate was loaded at its midplane using a single node to control force and displacement. Linear plane strain elements were used for substrate modeling, and periodic boundary conditions were applied to the lateral edges. Due to the system’s periodicity and symmetry, only half of the geometry was modeled, with the structures fixed at the lower edge in all case studies. (Fig 1).

**Fig 2:**
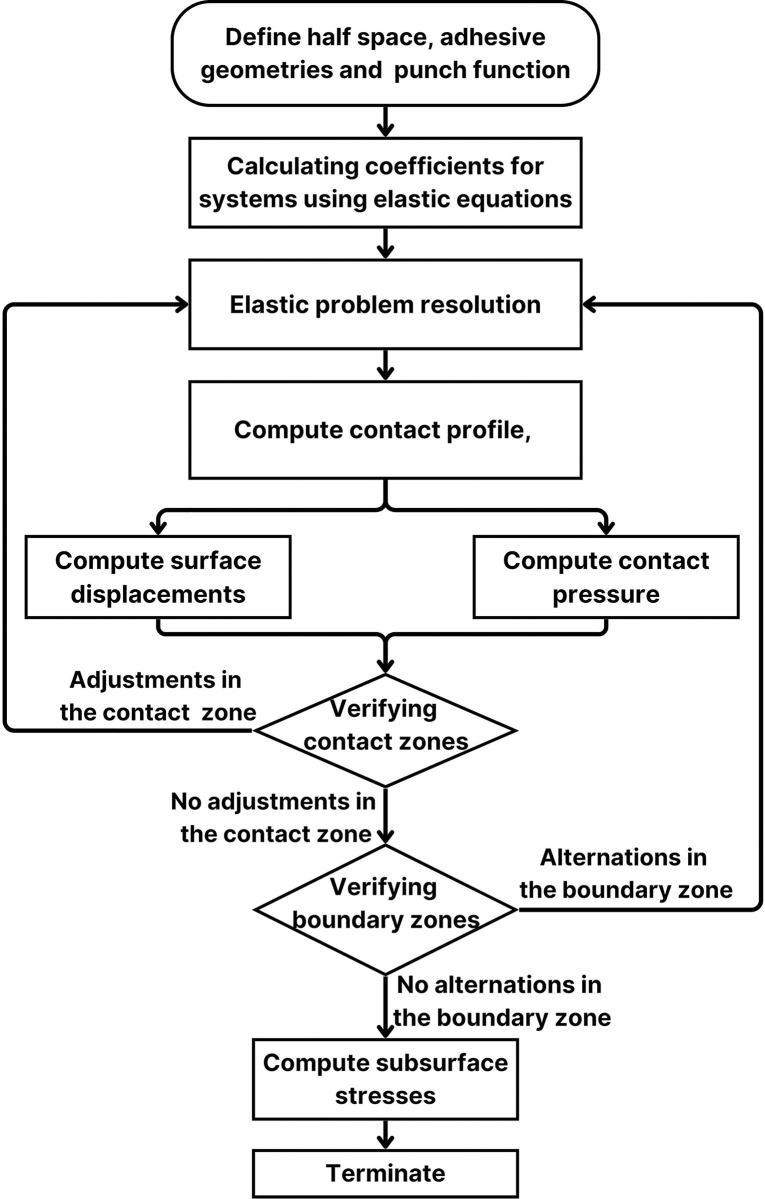
Mathematical modeling process for contact adhesion layer properties.

Fig 2 illustrates the mathematical modeling process used to analyze the impact on contact adhesion layer properties. The flowchart starts with the initial setup, including defining material properties and boundary conditions. It then progresses through the stages of numerical formulation, solution of governing equations, and validation of results. Each step is crucial for accurately capturing the behavior of the adhesion layer under various conditions, ultimately providing insights into its performance and characteristics in the simulated environment.

In this study, the punch geometry r(x) is defined by the function below, where Λ represents the rigid indenter amplitude and λ, the variable wavelength, ranges from 0.5, 2, and 4.


r(x)=Λcos(x/λ) (1)

E_adh_ and E_body_ denote the modulus of elasticity for the adhesive and the half body, respectively, with β being the ratio between them (elasticity ratio), The contact stiffness can be effectively increased by applying a stiffer layer or decreased by applying a softer layer to the body changing by 5/1, 2/1, 1/1, 0.5/1 and 0.1/1.


$$\beta =\frac{E_{adh}}{E_{body}}$$ (2)

Where μ_adh_, μ_body_ are the shear modulus and υ_adh_, υ_body_ are their Poisson’s ratio determinable for both adhesive and half-space body.


$$\mu_{adh}=\frac{E_{adh}}{2(1+v_{adh})},\mu_{body}=\frac{E_{body}}{2(1+v_{body})}$$ (3)

$E_{adh}^{*}$, $E_{body}^{*}$ represent the effective modulus of elasticity for the adhesive and the half body, respectively, with their ratio denoted as E^✽^.


$$E_{adh}^{*}=\frac{E_{adh}}{\left(1-v_{adh}^{2}\right)},E_{body}^{*}=\frac{E_{body}}{\left(1-v_{body}^{2}\right)}$$ (4)

$$E^{*}=\frac{E_{adh}^{*}}{E_{body}^{*}}$$ (5)

The relations of the linear theory of viscoelasticity [42] between tensor stresses and strains are expressed in the following form:

$$\sigma (t)=\int_{0}^{t}K(t-\tau )d\theta (\tau )$$

$$\theta (t)=\int_{0}^{t}\Pi_{\theta }(t-\tau )d\sigma (\tau )$$ (6)

$$S_{ij}(t)=\int_{0}^{t}2H(t-\tau )de_{ij}(\tau )$$

$$e_{ij}(t)=\int_{0}^{t}\Pi (t-\tau )dS_{ij}(\tau )$$

In equations (6), σ(t) is average stress, θ(t) volumetric deformation, and S_ij_(t), e_ij_(t) deviators of stress and strain tensors, K(t), 2H(t), volume and shear relaxation kernels, Π_θ_(t), Π(t) cores of volumetric and shear creep. After the application of integral Laplace-Carson transforms these relations. After the application of integral Laplace-Carson transforms these relations.


$$f^{*}(s)=s\overset{\infty }{\underset{0}{\int }}e^{-st}f(t)dt$$ (7)

Here f(t) represents the function in its original domain before undergoing the transformation, the increase of which t→∞ does not exceed the decrease of the exponential function e^-γt^, γ>0, f^✽^(s) transform functions f(t) in the Laplace–Carson transform s is a positive real variable. Taking into account the known properties of the Laplace–Carson transformation, the expressions for stresses and strains in the defining relations will take the form:

$$\sigma^{*}(s)=K^{*}(s)\theta^{*}(s)$$

$$\theta^{*}(s)=\Pi_{\theta }^{*}(s)\sigma^{*}(s)$$ (8)

$$S_{ij}^{*}(s)=2H^{*}(s)e_{ij}^{*}(s)$$

$$e_{ij}^{*}(s)=\Pi^{*}(s)s_{ij}^{*}(s)$$

Exploring the correlation between the logarithm of the surface response function S(q) is a corrective factor that takes into account the system geometry and boundary conditions. It is used in the context of layered solids to determine the specific form of the term representing the interfacial normal stresses and surface normal displacements in the system. This investigation encompasses scenarios where a stiff layer overlies a soft semi-infinite solid and where a soft layer coats a semi-infinite stiff solid. m and n are parameters depending on the material properties and geometry [43].


$$S(q)=\frac{1+4mqde^{-2qd}-mne^{-4qd}}{1-(m+n+4mq^{2}d^{2})e^{-4qd}+mne^{-4qd}}$$ (9)

Where d denotes the layer thickness and q is the wavenumber related to the spatial frequency of the punch shape. s only depends on qd, υ_adh_, υ_body_, E_adh,_ and E_body_ it is dimensionless.


$$m=\frac{\eta -1}{\eta +3-4v_{adh}},n=1-\frac{4(1-v_{adh})}{1+\eta (3-4v_{body})}$$ (10)

Where η is the ratio between the adhesive and half-space shear modulus.


$$\eta =\frac{\mu_{adh}}{\mu_{body}}$$ (11)

The relationship between deformations ε_ij_ and displacements u_i.j_ and u_j,i_ are established as below:

$$\varepsilon_{ij}=(u_{i,j}+u_{j,i})/2$$ (12)

Using the Somigliana formulas [44] obtained in the theory of elasticity, The transformation of the desired functions can be written in the form:

$$u_{i}^{*}(s)=\lambda^{*}(s)G_{i}(2H^{*}(s),K^{*}(s),\Pi^{*}(s),\Pi_{\theta }^{*}(s),x,m)+\mu^{*}(s)p_{i}(2H^{*}(s),K^{*}(s),\Pi^{*}(s),\Pi_{\theta }^{*}(s),x,m)$$ (13)

In the formula for u_i_^✽^(s) x coordinates of a body point and from formula (13), λ^✽^(s) is the equalities follow, where is the wavelength and p_i_ denote the local contact pressure, the following equalities hold:

$$2H^{*}(s)\Pi^{*}(s)=1,K^{*}(s)\Pi_{\theta }^{*}(s)=1$$ (14)

Taking into account these dependencies between 2G^✽^(s) and Π^✽^(s), the expression ui✽(s) can be represented in the form:

$$u_{i}^{*}(s)=\lambda^{*}(s)G_{i}(2H^{*}(s))+\mu^{*}(s)p_{i}(2H^{*}(s))$$ (15)

This expression omits all arguments that do not depend on the parameter s. This means, in particular, that the boundary conditions are written relative to the geometry of the body, which does not depend on time. These functions can be approximated in image space in different ways. One of them is the representation of these functions by segments of the Laurent series. As an example, consider this representation for the function G_i_(2H^✽^(s)) with the required accuracy in image space, and then construct its original in the form.


$$G_{i}(2H^{*}(s))=a_{i}+b_{i}2H^{*}(s)+c_{i}\frac{1}{2H^{*}(s)}+\ldots$$ (16)

Having found the required coefficients a_i_, b_i_, c_i_, … approximations of the function Gi(2H✽(s)) can be achieved with the required accuracy in space transformation, G_λ_(x) is the layered periodic Green’s function and then build its original in the below statement:

$$G_{\lambda }(x)=a_{i}+b_{i}2G(t)+c_{i}\Pi (t)+\ldots$$ (17)

For linear materials, surface displacements u_y_(x) can be expressed as below and G_λ_(x-s) is Green’s function representing the displacement response at x due to a point load applied at s. Λ is the rigid indenter amplitude, distributed load over the contact domain of integration Ω has been shown by p(s).


$$u_{y}(x)=-\int_{\Omega }G_{\lambda }(x-s)p(s)ds=\Delta +r(x)-\Lambda$$ (18)

Δ denotes contact penetration, defined as the distance between the deformed surface mean plane (u_T_) and the peaks of the sinusoidal indenter (u_m_).


$$\Delta =u_{T}-u_{m}$$ (19)

Consequently, u(q) is the wavevector domain’s linear response function, the linear response function is shown by M_zz_(q) and the stress field in the wavevector domain is shown by σ(q), and the wavevector is shown by q.


$$u(q)=M_{zz}(q)\sigma (q)$$ (20)

The specific form of M_zz_(q) depends on the system geometry, the material properties, and how the system is constrained.


$$M_{zz}(q)=-\frac{2(1-v_{body}^{2}}{E_{body}}\frac{1}{q}S(q)$$ (21)

S(q) is the corrective factor taking into account the system geometry and boundary conditions. Notice in the case of homogenous elastic half-space. P illustrates the contact pressure within the contact domain of integration where the load is applied (Ω), and the λ is the variable wavelength.


$$P=\lambda^{-1}\int_{\Omega }p(x)dx$$ (22)

The general equations that define the mathematical model of the problem under consideration can be represented in the form of equilibrium equations:

$$\sigma_{ij,j}=X_{i}^{0}(x)\psi (t)$$ (23)

The boundary condition is:

$$\sigma_{ij}l_{j}{|}_{\sum_{1}}=\sigma_{i}^{0}(x)\psi (t)$$ (24)

$$u_{i}{|}_{\sum_{2}}=u_{i}^{0}\mu (t)$$ (25)

Where X_i_ are volumetric forces, Cauchy relations for small deformations, ψ(t) is the stress relaxation shear modulus. The interfacial elastic energy stored in the elastic material is denoted as ε(a) [45]. It is calculated using the contact pressure distribution and the displacement field of the elastic material.


$$\varepsilon (a)=\frac{1}{2}\int_{D}p(x)u_{y}(x)dx=\frac{1}{2}\int_{D}p(x)[r(x)-\Lambda +\Delta ]dx$$ (26)

$$\frac{1}{K_{s}(a)}=\frac{1}{f_{1}(a)K_{adh}}+\frac{1}{f_{2}(a)K_{body}}$$ (27)

K_s_(a) is the global stiffness of the system, and K_l_, K_h_ are the stiffnesses of the adhesive layer and the half-space, f_1_(a), f_2_(a) are functions that depend on the system geometry and the parameter a.


$$K_{adh}=\frac{aB}{d}\frac{E_{adh}}{1-\upsilon_{adh}^{2}}$$ (28)

B is the width dimension, d is the thickness of the adhesive layer, and as mentioned above ν is the Poisson’s ratio of the adhesive layer.

## Parametric analyses

The paper explores several parametric variables to comprehensively understand their influence on the mechanical behavior of the coated structure. These variables include variations in the radius of the punch, the relationship between the modulus of elasticity of the adhesive and the elastic body, and changes in the thickness of the adhesive layer. The thickness of the elastic body, which is considered to have a finite value in this study unless otherwise stated, can be finite in the context of the Finite Element Method.

**Fig 3:**
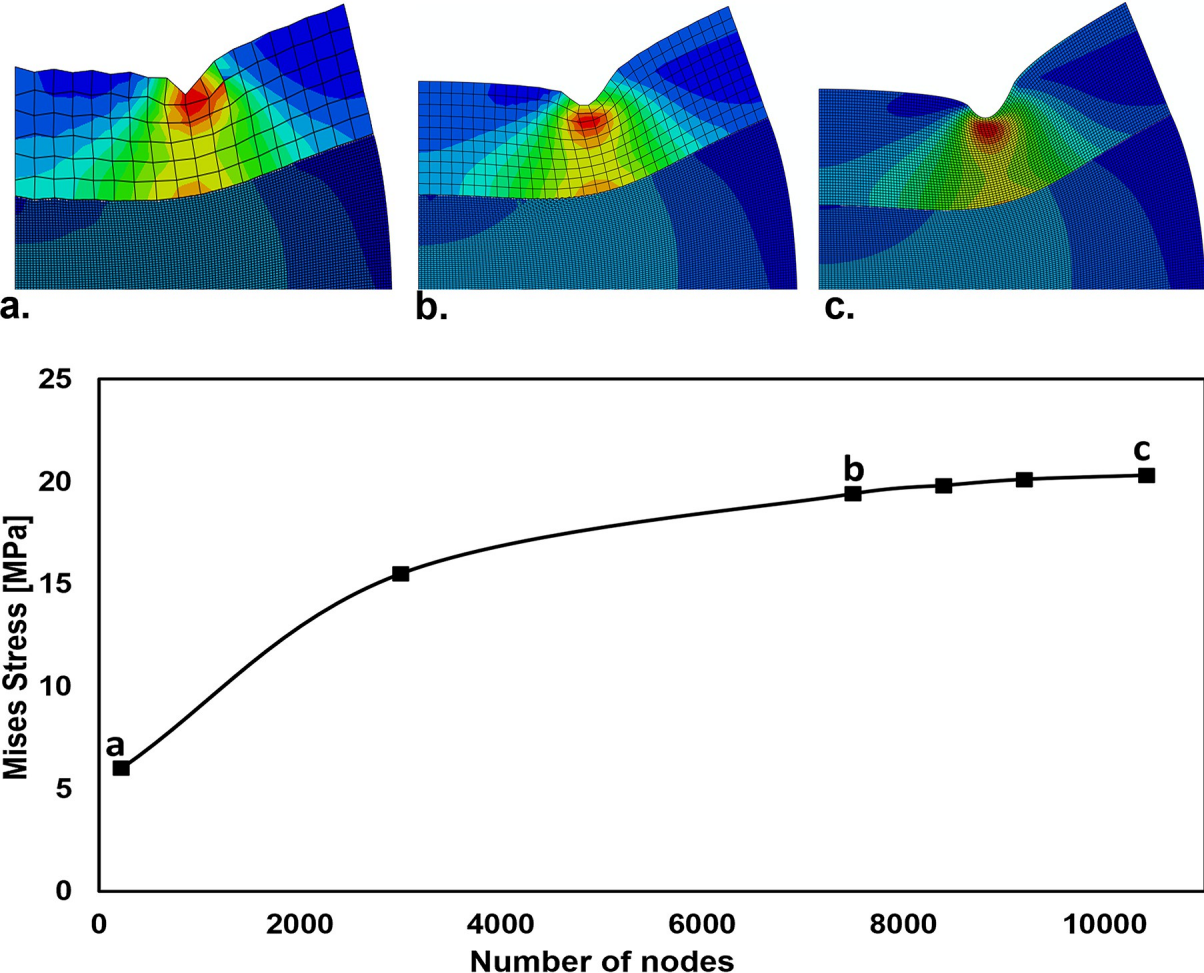
Upper: Finite element models with varying mesh sizes (a. 0.4 mm, b. 0.1 mm, c. 0.05 mm) and Mises stress distribution. Bottom: Showing the relationship between mesh size and Mises stress distribution across six case studies.

Fig 3 the upper image: These models visually demonstrate the impact of mesh size on the accuracy of numerical simulations. The analyses show that the highest precision is achieved with a 0.05 mm mesh size, emphasizing the importance of element size in obtaining accurate results. The finer mesh better captures the complexities of interfacial penetration and contact pressure dynamics, offering more reliable insights into the adhesive layer’s mechanical behavior. Fig 3 the bottom image: This graph highlights the error in Mises stress across six models. For example, the most accurate model, with a mesh size of 0.05 mm and 10420 nodes, compared to the second most accurate model with 9220 nodes, shows a 0.8% error in Mises stress. Based on this minimal error, the finer model has been selected for the numerical simulation analysis.

**Fig 4:**
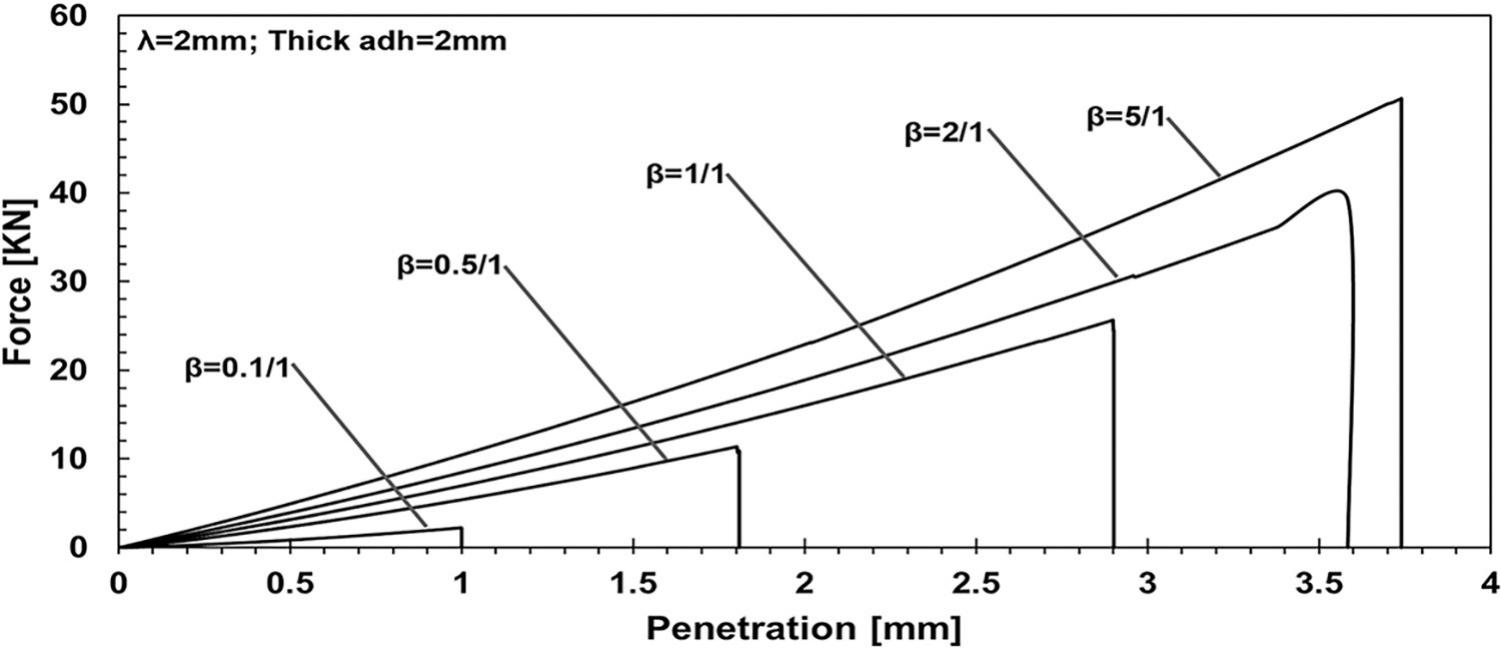
Quantifying the effect of Young’s modulus ratio on force-penetration profiles: Adhesive layer thickness maintained at 2 mm, wavelength fixed at 2.

In a series of force-penetration profiles obtained from numerical simulations (Fig 4), the influence of the varying Young’s modulus ratio on the penetration force exerted by a punch onto an adhesive layer is observed. The adhesive layer thickness is kept constant at 2 mm, and the wavelength of the applied load is fixed at 2 units. Each curve represents a different ratio of elastic moduli between the adhesive and the substrate.

**Fig 5:**
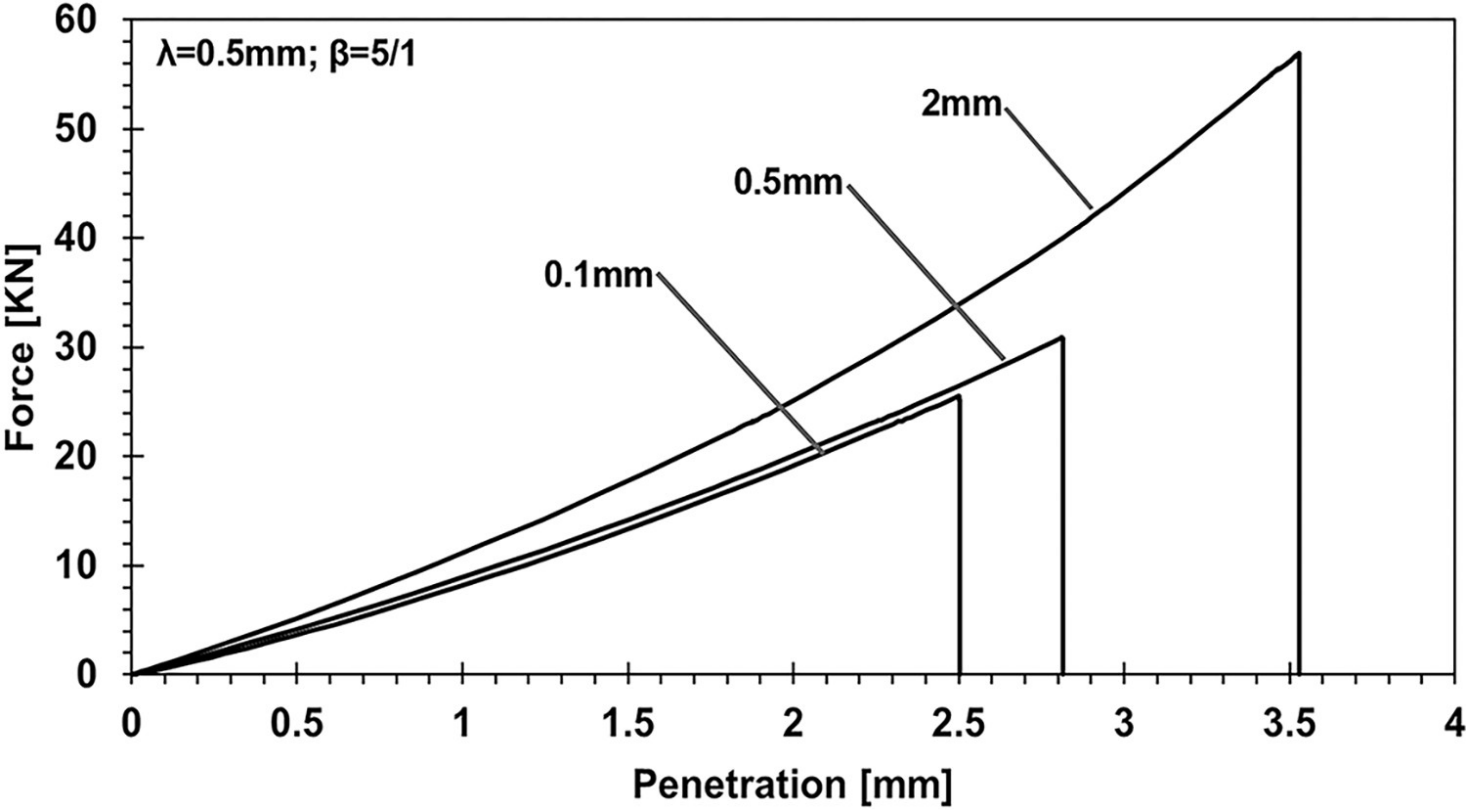
Exploring the influence of adhesive layer thickness on force-penetration behavior: Consistent wavelength of 0.5 and Young’s modulus ratio fixed at 5/1.

Fig 5 analyzes the effects of varying adhesive layer thickness on the relationship between applied load and punch penetration. With the λ punch parameter fixed at 0.5mm and a coating-to-half-body stiffness ratio of 5:1, the case studies compare adhesive layer thicknesses of 0.1mm, 0.5mm, and 2mm. The findings highlight significant differences in load-penetration behavior across the different adhesive layer thicknesses, demonstrating how variations in layer thickness impact the mechanical response under applied load.

**Fig 6:**
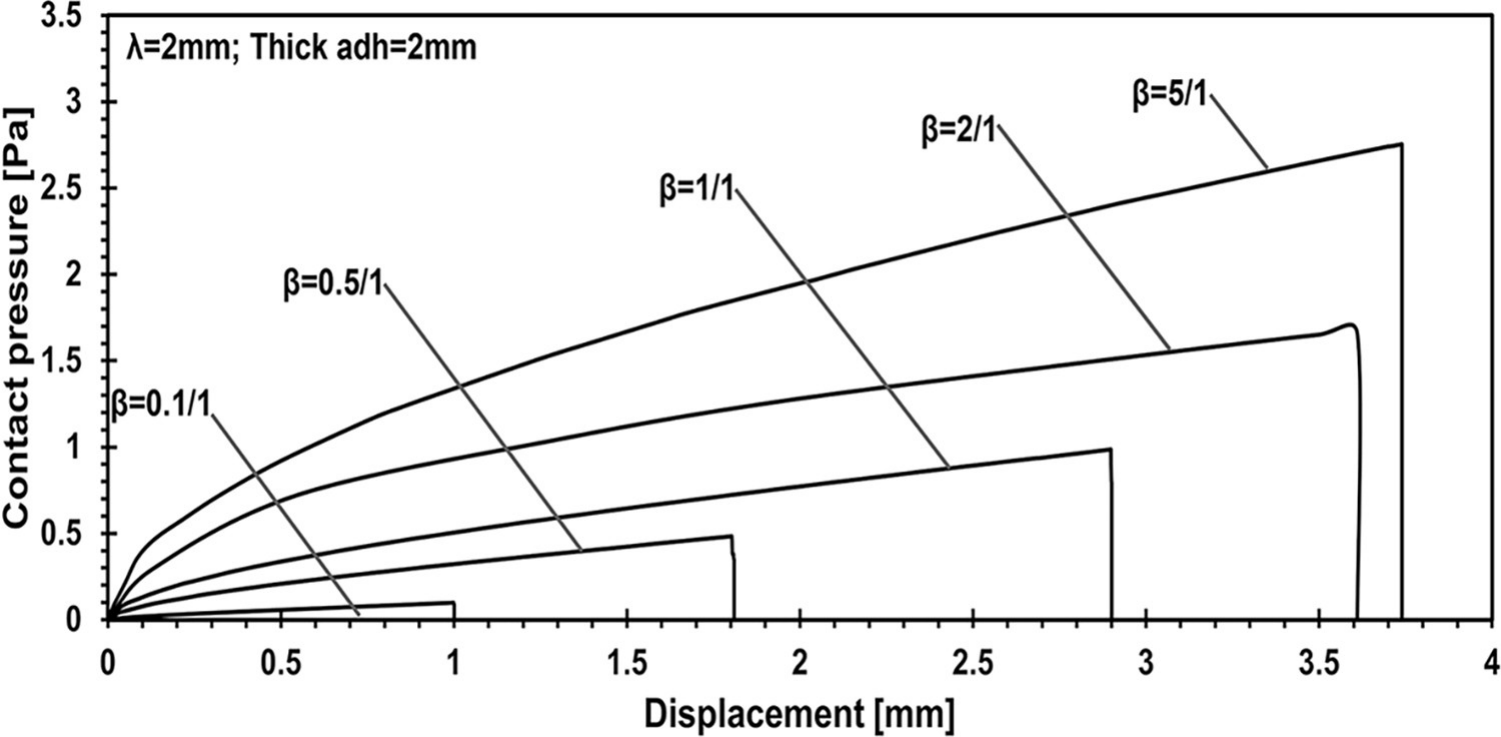
Quantifying the effect of Young’s modulus ratio on contact pressure-displacement profiles: Adhesive layer thickness maintained at 2 mm, wavelength fixed at 2.

The relationship between Young’s modulus ratio and contact pressure-displacement profiles is the focus of analysis in Fig 6. Here, the contact pressure-displacement curves depict how altering the ratio of elastic moduli between the adhesive and the substrate influences the pressure distribution as the punch penetrates the adhesive layer. The adhesive layer thickness is maintained at 2 mm, and the wavelength of the applied load remains fixed at 2 units, allowing for a detailed examination of how material properties affect the mechanical response of the adhesive system.

**Fig 7:**
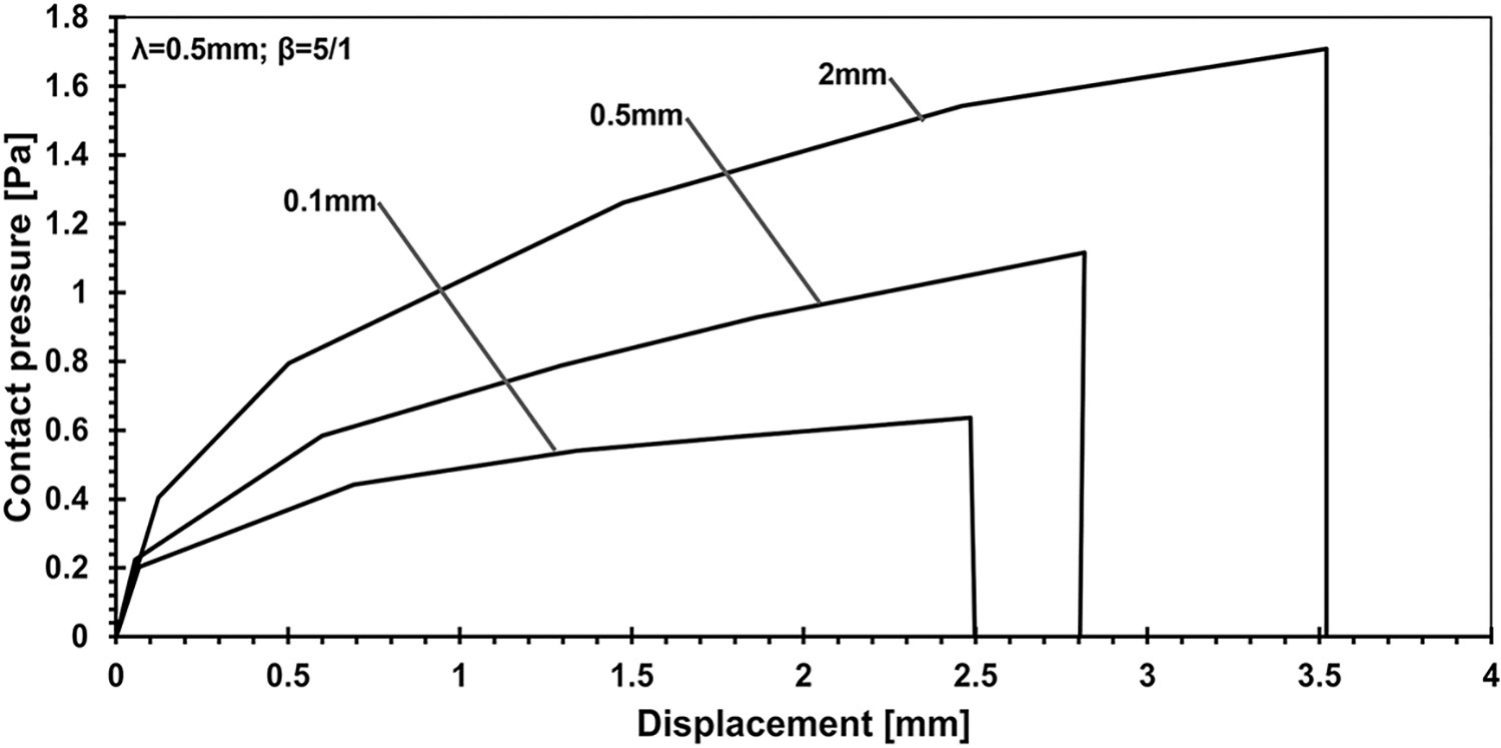
Exploring the influence of adhesive layer thickness on contact pressure-displacement behavior: Consistent wavelength of 0.5 and Young’s modulus ratio fixed at 5/1.

In Fig 7, the influence of adhesive layer thickness variations on contact pressure-displacement behavior is investigated. By keeping a consistent wavelength of 0.5 and a fixed Young’s modulus ratio of 5/1, the study examines how changes in adhesive thickness (0.1, 0.5, and 2 mm) impact the distribution and magnitude of contact pressure as the punch interacts with the adhesive layer. These findings offer valuable insights into the role of adhesive geometry in determining the mechanical behavior of adhesive systems under external loading conditions.

**Fig 8:**
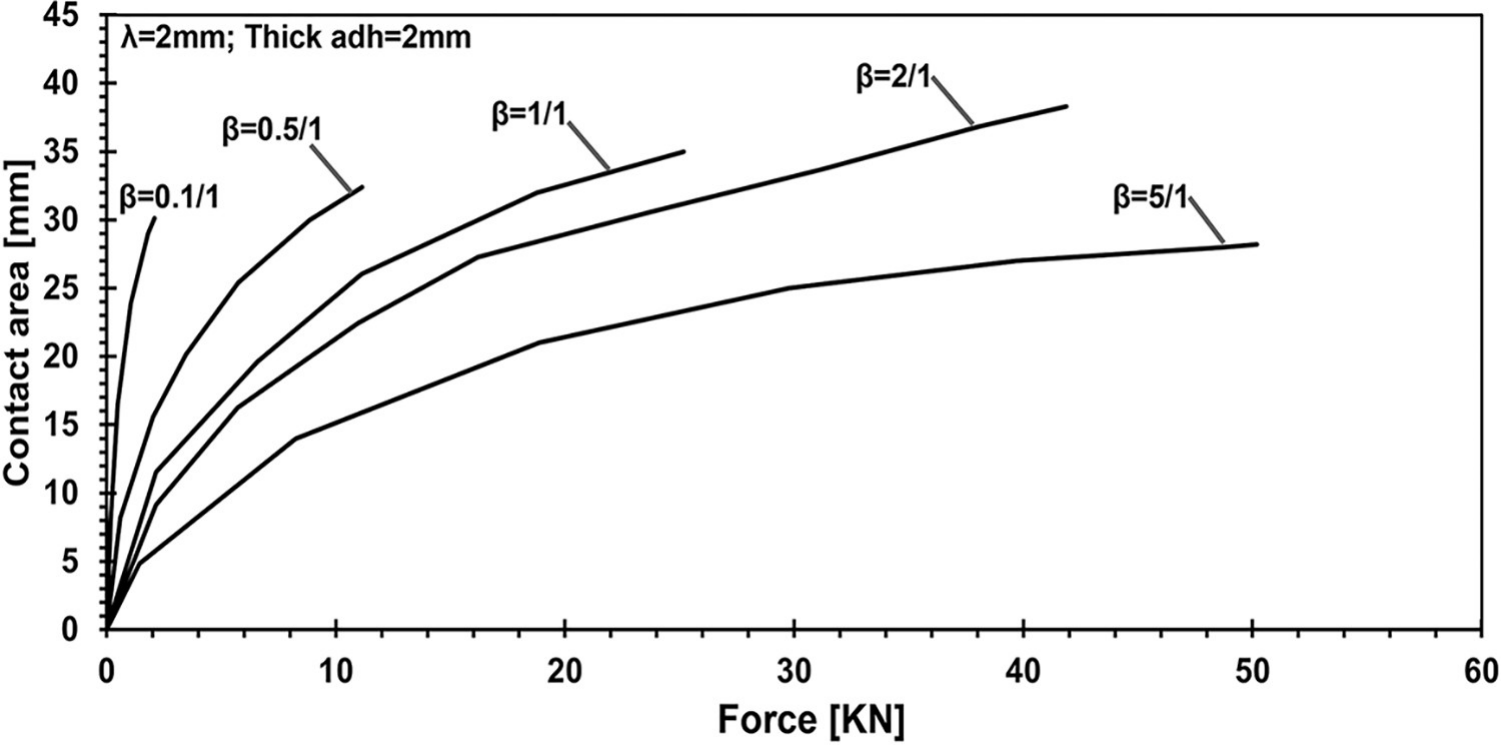
Quantifying the effect of Young’s modulus ratio on contact area- applied force profiles: Adhesive layer thickness maintained at 2 mm, wavelength fixed at 2.

In the study, Fig 8 quantifies the effect of different Young’s modulus ratios on contact area-applied force profiles, ranging from the softest adhesive layer (0.1/1) to the hardest model. By fixing the adhesive layer thickness at 2 mm and the wavelength of the applied load at 2 units, the research explores how variations in the ratio of elastic moduli between the adhesive and the substrate influence both the contact area and the magnitude of applied force.

**Fig 9:**
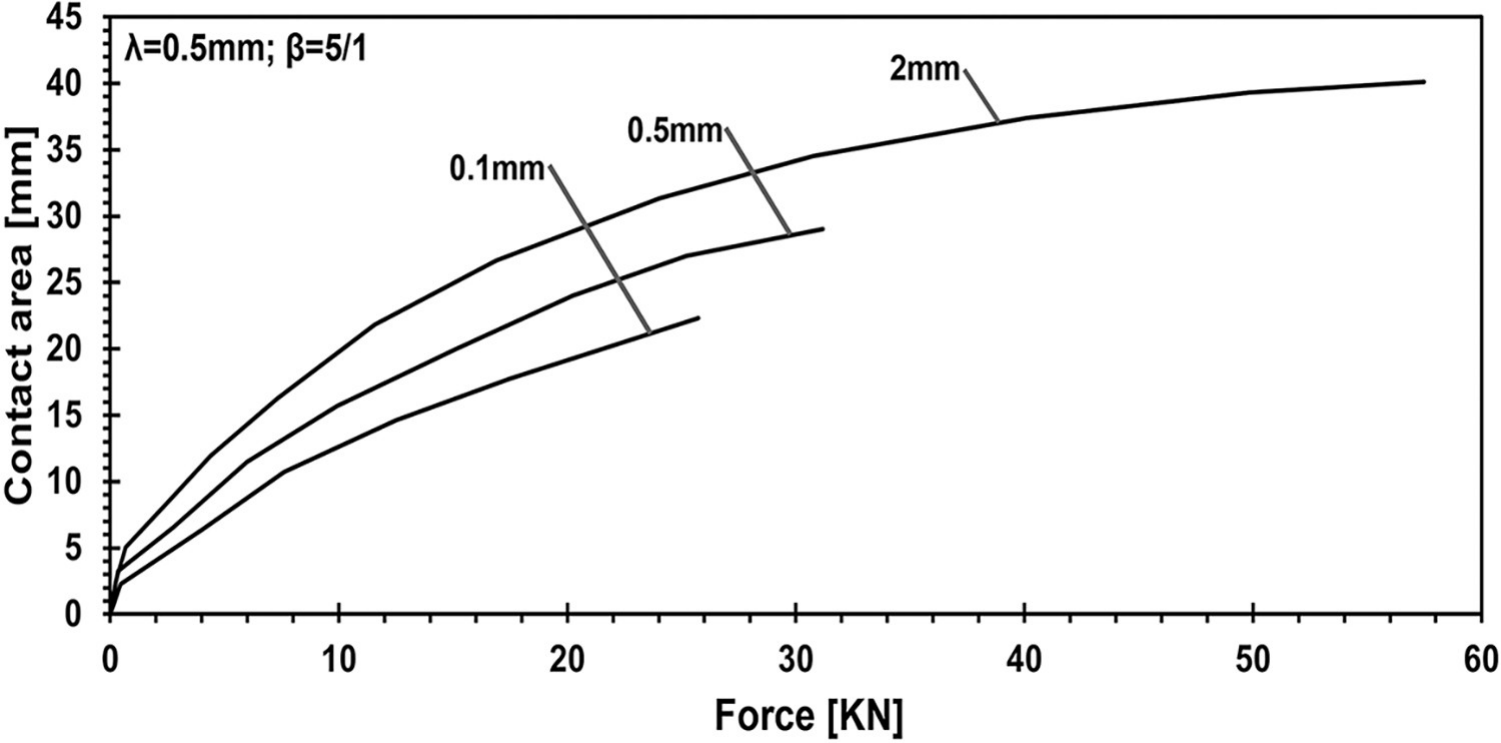
Exploring the influence of adhesive layer thickness on contact area- applied force behavior: Consistent wavelength of 0.5 and Young’s modulus ratio fixed at 5/1.

Exploring the influence of adhesive layer thickness on contact area-applied force behavior is depicted in Fig 9, ranging from a thin adhesive layer (0.1 mm) to a thicker coated material (2 mm). With a consistent wavelength of 0.5 and a fixed Young’s modulus ratio of 5/1.

**Fig 10:**
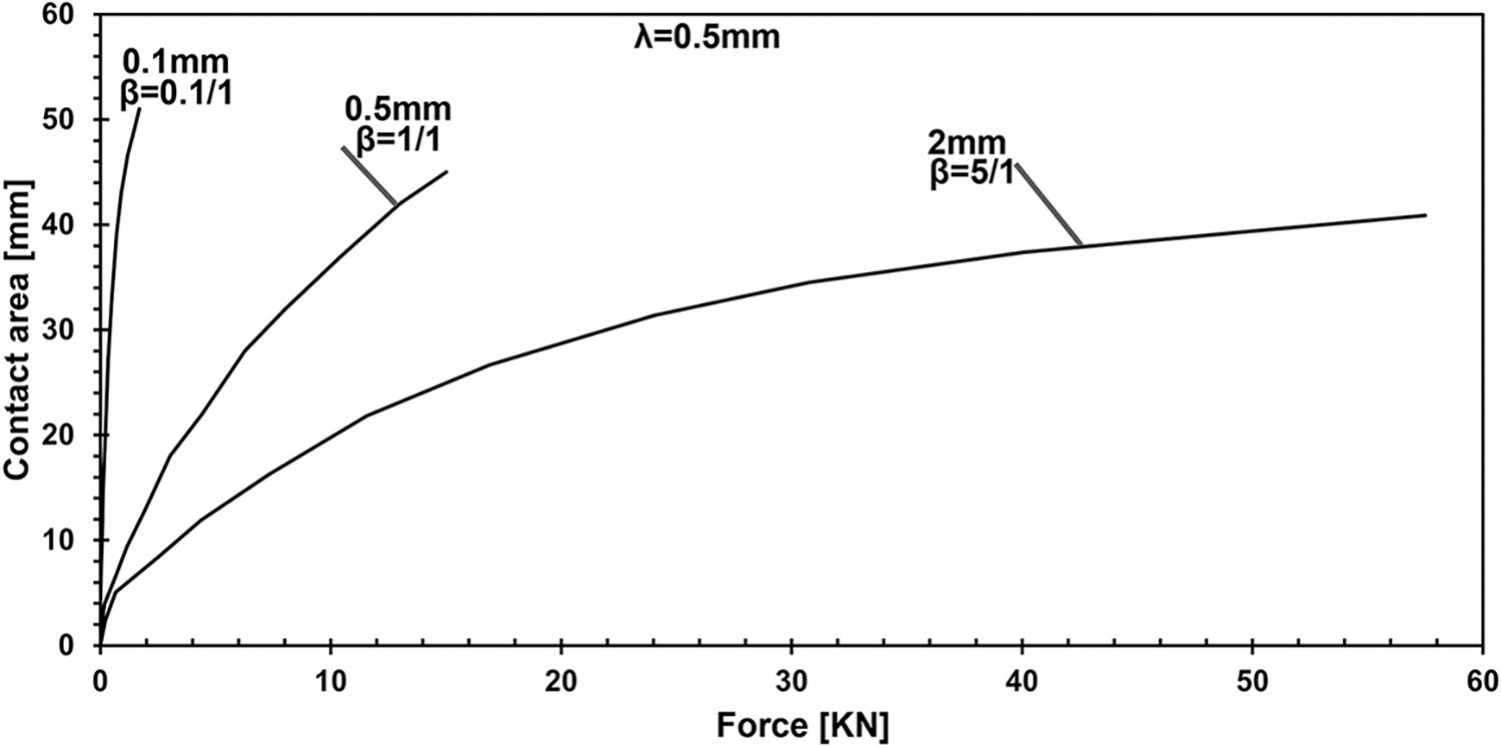
Quantifying the influence of Young’s modulus ratio and adhesive layer thickness on force-contact area.

Investigating three distinct scenarios, Fig 10 portrays a thin adhesive layer with a low modulus of elasticity ratio, a moderate thickness adhesive layer with a balanced ratio, and a thick adhesive layer with a high modulus of elasticity ratio. Through these cases, the figure offers insights into how variations in adhesive layer thickness and modulus of elasticity ratio influence force-contact area, contributing to the comprehension of contact adhesion layer properties in numerical simulations.

When comparing our study on contact adhesion layer properties with another significant paper in this field [41] key distinctions and similarities arise. Our research focuses on adhesive layer thickness, elasticity modulus, and punch geometry, revealing relationships between contact stiffness, material flexibility, and contact area. In contrast, the referenced study emphasizes viscoelastic behavior, addressing transient responses and surface roughness effects. Both studies use numerical methods for validation, highlighting the influence of material properties and geometry in contact mechanics. Our work enhances the understanding of elastic contact mechanics and adhesive optimization, while the viscoelastic study provides insights into polymer behavior under complex loading, demonstrating the broad applicability of contact mechanics principles.

In the case where the adhesive layer thickness is set to 2 mm and the lambda parameter is fixed, the effect of varying the elasticity ratio, beta, was analyzed. The results indicate that the difference in penetration between the stiffest configuration (5:1 ratio) and the softest (0.1:1 ratio) is 3.74 times. Furthermore, the applied force increases by 22.96 times, and contact pressure rises by 28.11 times. However, an important observation is that the contact area decreases by 7%. The highest contact area, measuring 38.28 mm, was achieved with a beta ratio of 2:1, highlighting the balance between stiffness and contact performance. It has been illustrated that the thickness does not have the most significant influence over the results.

On the other hand, another analysis explored the effect of varying adhesive layer thickness while keeping the lambda parameter at 0.5 mm and the elasticity ratio fixed at 5:1. When altering the adhesive thickness from 0.1 mm to 2 mm, the results show increases in penetration by 1.41 times, applied force by 2.23 times, contact pressure by 2.68 times, and contact area by 1.8 times. These findings suggest that simply making the adhesive layer stiffer does not necessarily lead to an increase in the contact area. Instead, achieving optimal mechanical behavior requires balancing the adhesive layer thickness, modulus of elasticity, and punch geometry.

## Conclusion

This study employed numerical simulations to investigate the contact mechanics of elastic half-planes with varying adhesive layer thicknesses, focusing on key parametric influences such as the punch radius, adhesive elasticity modulus, and layer thickness. These analyses explored the relationship between the mechanical behavior of the adhesive system and these variables, providing deeper insights into surface interaction. Our findings reveal a direct relationship between contact stiffness and the flexibility contrast between coating and substrate materials. As demonstrated in the parametric analysis (Fig 5), changes in the adhesive layer thickness and Young’s modulus ratio significantly affect the load-penetration and pressure-displacement profiles. For instance, it was observed that thinner adhesive layers exhibit greater mechanical flexibility, requiring softer materials to achieve full adhesion, while thicker layers increase load-bearing capacity but reduce contact area. Specifically, increasing the adhesive layer thickness from 0.1 mm to 2 mm leads to a 2.23-fold rise in load-bearing capacity, as highlighted in the quantitative analysis. Additionally, the study shows that targeted modifications to coating parameters, such as adhesive layer thickness and punch geometry, can induce full contact conditions with minimal external force. For example, transitioning from a wide punch to a narrower one reduced penetration by 5.67% and contact pressure by 37.98%, while expanding the contact area by 42.2%, as depicted in Figs 6 and 7. The relationship between the adhesive layer thickness and the resulting contact area was also explored in detail (Fig 10). A 0.1. Furthermore, it was illustrated that increasing the stiffness of the adhesive layer does not guarantee an increase in contact area or performance. Instead, there is a need to maintain a balance between the adhesive layer thickness, its modulus of elasticity, and punch geometry to achieve optimal mechanical behavior. These observations underscore the critical role of material flexibility in achieving optimal contact conditions between the substrate and rigid counterparts.

## Supporting information

S1 Data (XLSX)
